# Assessing the validity of inertial measurement units for shoulder kinematics using a commercial sensor‐software system: A validation study

**DOI:** 10.1002/hsr2.772

**Published:** 2022-08-10

**Authors:** Jakob Henschke, Hannes Kaplick, Monique Wochatz, Tilman Engel

**Affiliations:** ^1^ Department for sports medicine and sports orthopedics, University Outpatient Clinic University of Potsdam Potsdam Germany

**Keywords:** diagnostic techniques and procedures, kinematics, shoulder joint, validation study, wearable devices

## Abstract

**Background and  Aims:**

Wearable inertial sensors may offer additional kinematic parameters of the shoulder compared to traditional instruments such as goniometers when elaborate and time‐consuming data processing procedures are undertaken. However, in clinical practice simple‐real time motion analysis is required to improve clinical reasoning. Therefore, the aim was to assess the criterion validity between a portable “off‐the‐shelf” sensor‐software system (IMU) and optical motion (Mocap) for measuring kinematic parameters during active shoulder movements.

**Methods:**

24 healthy participants (9 female, 15 male, age 29 ± 4 years, height 177 ± 11 cm, weight 73 ± 14 kg) were included. Range of motion (ROM), total range of motion (TROM), peak and mean angular velocity of both systems were assessed during simple (abduction/adduction, horizontal flexion/horizontal extension, vertical flexion/extension, and external/internal rotation) and complex shoulder movements. Criterion validity was determined using intraclass‐correlation coefficients (ICC), root mean square error (RMSE) and Bland and Altmann analysis (bias; upper and lower limits of agreement).

**Results:**

ROM and TROM analysis revealed inconsistent validity during simple (ICC: 0.040−0.733, RMSE: 9.7°−20.3°, bias: 1.2°−50.7°) and insufficient agreement during complex shoulder movements (ICC: 0.104−0.453, RMSE: 10.1°−23.3°, bias: 1.0°−55.9°). Peak angular velocity (ICC: 0.202−0.865, RMSE: 14.6°/s−26.7°/s, bias: 10.2°/s−29.9°/s) and mean angular velocity (ICC: 0.019‐0.786, RMSE:6.1°/s−34.2°/s, bias: 1.6°/s−27.8°/s) were inconsistent.

**Conclusions:**

The “off‐the‐shelf” sensor‐software system showed overall insufficient agreement with the gold standard. Further development of commercial IMU‐software‐solutions may increase measurement accuracy and permit their integration into everyday clinical practice.

## 1 INTRODUCTION

The assessment of upper limb function has become a viable tool in clinical decision making for professions in the medical and athletic field. In overhead sports, the monitoring of shoulder range of motion (ROM) has been emphasized, to distinguish between physiological adaptation and maladaptation.^1^, ^2^ Due to the excessive forces and repetitive loading of the shoulder complex, limited internal rotation (IR) and increased external rotation (ER) ROM was observed when comparing dominant and nondominant shoulders of baseball players in 90° abduction (ABD).^3^ Similar side‐to side differences were evident during shoulder vertical flexion (VFLEX), horizontal flexion (HFLEX) and total range of rotational motion, which is defined as the total arc of ER and IR.^3^, ^4^, ^5^ These adaptations may serve clinicians and trainers as potential predictors for future injuries in overhead athletes.^6^, ^7^, ^8^, ^9^ Additional kinematic parameters such as angular velocities may be utilized as indicators for movement smoothness in clinical rehabilitation.^10^, ^11^, ^12^, ^13^

Traditional instruments to evaluate shoulder kinematics include digital or analog goniometers as well as gravity‐based inclinometers. However, goniometers are prone to in accuracy, since the obtained angles rely on the type of joint and movement, investigators experience, and patient positioning.^14^, ^15^, ^16^ Furthermore, they are limited to static measurement conditions while only delivering ROM output without additional kinematic parameters.

On the other hand, camera‐based motion capture (Mocap) is still referenced as the gold standard for kinematic assessments of upper limb segments due to its high accuracy.^17^, ^18^, ^19^, ^20^, ^21^, ^22^ Yet, these systems may not be suitable for clinical practice since they require a large amount of preparation time, experienced operators, and a laboratory environment to achieve valid results. A promising alternative to objectively quantify body kinematics are portable devices such as inertial measurement units (IMU).^23^, ^24^ Major advantages of these sensors compared to the gold standard are relative cost‐effectiveness, reduced time investment and the capability to extract real‐time data. IMU sensors are valid instruments which can be applied in different field‐applications such as clinical or scientific movement analysis, monitoring of activities of daily living as well as sports performance assessment.^25^, ^26^, ^27^ Some specific examples may be the assessment of postural sway,^28^ gait analysis,^29^, ^30^, ^31^ the evaluation of jumping characteristics,^32^ fall detection.^33^, ^34^

Concerning upper limb kinematics, Morrow et al. found an accuracy of shoulder flexion angles up to 7° during a simulated surgery compared to the gold standard.^35^ Similarly, Poitras et al. were able to show an average error during shoulder elevation of ≤10.2°, while reporting similar results for a complex lifting task.^18^, ^20^, ^36^ Yet, a substantial variance of validity was found between studies in a current systematic review by Walmsley et al. with ranges between 0.1° and 15.0° for upper limb ROM.^37^ The authors concluded that validity tends to decrease with increasing task complexity.^35^, ^36^, ^37^

Although these results indicate sufficient validity, most investigations utilized highly customized software and fusion algorithms as well as adapted calibration methods. There is only limited evidence for well‐established technology transfer of IMU systems into the clinical field.^38^, ^39^

A primary reason for this is the elaborate and time‐consuming data processing procedures required (e.g., sensor set‐up and calibration, adaptation of sensor‐fusion algorithms, data export), which do not offer simple real‐time motion analysis. Therapists, trainers, and other professionals should be able to set‐up IMU systems in a time‐efficient manner, allowing for accurate data capture and generation of clinically relevant parameters. Therefore, such “off‐the‐shelf” solutions might be appropriate when quantifying therapy progress or obtaining real time feedback regarding individual movement quality. Without advanced technical knowledge and training of the investigators, the transfer of valid IMU measurement constructs into everyday clinical practice appears challenging.

Accordingly, this study aims to validate a commercial “off‐the‐shelf” IMU sensor‐software system for the assessment of active shoulder kinematics during single‐ and multiplanar movements, compared with three‐dimensional camera system. Sufficient criterion validity may be expected for kinematic parameters during single‐plane shoulder movements.

## 2 MATERIALS AND METHODS

### 2.1 Participants

24 asymptomatic participants (9 female, 15 male, age: 29 ± 4 years, height: 177 ± 11 cm, weight: 73 ± 14 kg) were included based on similar research in this field.^40^, ^41^, ^42^ The cohort was recruited from the university's campus and consisted of sedentary and recreationally active adults to imitate a broad population. Inclusion criteria were age of at least 18 years, the absence of any (acute or chronic) shoulder pain, as well as understanding, and signing the provided written informed consent. Ethical approval was given by the university's ethics review board (grant number: 74/2020).

### 2.2 Instrumentation

Kinematic data from two portable IMU sensors (Wave Track inertial system; Cometa Systems) were compared against a 10‐camera motion capture system (Vicon MX T10S; Vicon Motion Systems), which is based on the requirements of the Vicon upper limb model (3 degrees of freedom), which is an extended version of the Plugin‐gait model for upper limb modeling. IMU data output was generated automatically by a 6‐degree‐of freedom model utilizing a proprietary sensor‐fusion algorithm of the integrated gyroscopes, accelerometers, and magnetometers. The recommended calibration procedures were completed for each sensor type based on manufacturer guidelines. The following outcome parameters were extracted: ROM [°] was calculated by subtracting the range of each movements end position in relation to the range of the starting position. TROM [°], was defined as the sum of both terminal ranges of each motion in relation to the starting position. Furthermore, minimum and maximum peak angular velocities (PAV [°/s]) were extracted from single plane movements, whereas mean angular velocities (MAV [°/s]) were obtained for all conditions.

### 2.3 Preparations and testing procedure

**Figure 1:**
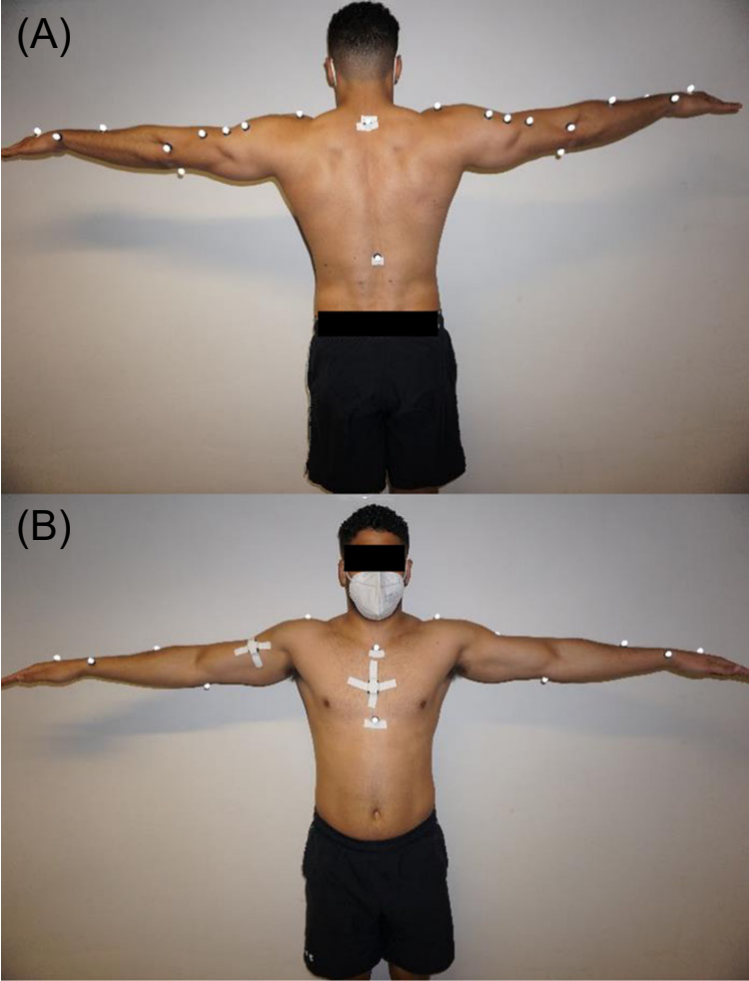
Calibration (“T‐pose”) position of the participant. (A) dorsal view, (B) frontal view. Reflective markers and two portable IMU sensors were attached on the torso. IMU sensor calibration pose. IMU, inertial measurement units.

Two portable IMU sensors were attached in a standardized way to the participants sternum (10 cm below the jugular fossa) and the frontal upper arm (15 cm below the acromion process) using rigid tape (Figure 1). Manufacturer guidelines only provided the broad requirements on placing the sensors on the upper arm and the sternum without more specific recommendations (User manual—EMG and Motion Tools v. 6.0). In addition, 23 retroreflective markers were attached to the participants torso and both arms with double layered tape based on the “upper limb model”.^21^ The participants were placed on a chair to minimize evasive movements of the trunk during the measurements. Before data assessment, the participants were asked to remain seated motionless in the neutral, anatomical position (trunk upright, arms, and hands lateral at the trunk) for calibration of the mocap system. Afterwards the “T‐pose” (arms 90° abducted) calibration pose for the IMU sensors was repeated before each measurement to align the sensor axes with the anatomical axis (Figure 1). Subsequently, single‐ and multiplanar movements were performed (movement characteristics are summarized in Table 1 and in Supporting Information: Figure 3 in Supporting Information: File 1). The arm to be tested was randomized, while each movement condition was performed once in a self‐selected comfortable velocity after one familiarization trial for each simple (abduction/adduction [ABD/ADD], vertical flexion/vertical extension [VFLEX/VEXT], horizontal flexion/horizontal extension [HFLEX/HEXT], external/internal rotation [ER/IR]) and complex [PNF = multiplanar shoulder‐elbow movement] shoulder movement.

**Table 1:** Movement characteristics and descriptions

Movement	Abbreviation	Plane	Starting position	End position
Abduction	ABD	Frontal	Arm relaxed lateral at the trunk (90° elbow flexion).	Arms maximally elevated lateral to the head (90° elbow flexion).
Adduction	ADD	Frontal	Arm relaxed lateral at the trunk (90° elbow flexion).	Arm passed by ventrally toward the sternum (90° elbow flexion).
Horizontal flexion	HFLEX	Transverse	Arm 90° abducted (90° elbow flexion).	Arm shifted towards the opposite shoulder while maintaining it parallel to the floor (90° elbow flexion).
Horizontal extension	HEXT	Transverse	Arm 90° abducted (90° elbow flexion).	Arm shifted backwards while maintaining it parallel to the floor (90° elbow flexion).
Vertical flexion	VFLEX	Sagittal	Arm relaxed lateral at the trunk (elbow extended).	Arm elevation to the front (elbow extension).
Vertical extension	VEXT	Sagittal	Arm relaxed next to the trunk (elbow extended).	Elevating the arm backwards (elbow extension).
External rotation	ER	Transverse	Arm relaxed lateral at the trunk (90° elbow flexion).	Rotation of the lower arm outwards while maintaining it parallel to the floor (90° elbow flexion).
Internal rotation	IR	Transverse	Arm relaxed lateral at the trunk (90° elbow flexion).	Moving the lower arm inwards while maintaining it parallel to the floor (90° elbow flexion).
Complex arm pattern	PNF	Frontal/transverse/sagittal	Arm placed at the opposite pelvic crest (shoulder in VFLEX/ADD/IR; elbow flexion).	Arm extended next to the head (shoulder in VEXT/ABD/ER; elbow extension).

Abbreviations: ABD, abduction; ADD, adduction; ER, external rotation; HFLEX, horizontal flexion, HEXT, horizontal extension; IR, internal rotation; PNF, a complex movement pattern; VFLEX, vertical flexion; VEXT, vertical extension.

### 2.4 Data processing and data reduction

**Figure 2:**
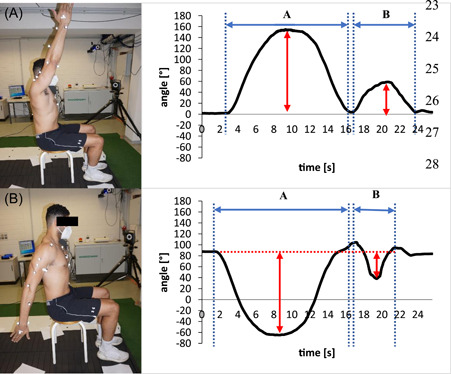
Left: Participant performing Vertical flexion (A) and vertical extension (B). Right: angle‐time plots for Mocap (top: the red arrow indicates the calculation of a maximum) and IMU (bottom: the red arrow indicates the calculation of minimum) movement fragmentation (blue arrows indicate beginning/end of movement) before angle offset removal. Vertical flexion and vertical extension with corresponding time‐angle plots. IMU, inertial measurement units.

Kinematic IMU data was collected and processed using an “off‐the‐shelf” software (EMG and Motion Tools; Version: 7.2.2.). Based on the displacement of the attached two IMU sensors (represented by thorax and upper arm) the resulting Euler angles were generated using the pre‐defined sensor‐position mapping as required in the “off‐the‐shelf” software. The ROM parameters were reported visually as time‐angle plots for “ABD/ADD,” “VFLEX/VEXT,” and “HFLEX/HEXT” in the software without further data processing. Angular velocities were based on the  gyroscope data from XZ′Y″ Euler angle components which corresponds to the ROM parameter definitions which were not presented instantly in the software interface but made available after data export. For detailed analysis, all kinematic data was exported as ASCII files and later processed in a table‐calculation software (Microsoft Excel; Version 365). In the table calculation software, time‐angle curves were plotted to define the outward movement (movement initiation and return to starting position), as well as the reverse movement (movement initiation and return to terminal position) for all outcomes (Figure 2) except MAV. MAV was calculated using narrower time windows instantaneously after initiation and shortly before reaching terminal position of both outward and reverse movements. This movement fragmentation allowed for minimum and maximum calculations for ROM/PAV and removing potential angle offsets retrospectively. As MAV may be the most valuable indicator for the assessment of movement quality, PAV was not calculated for ER/IR and PNF. Additionally, MAV was chosen as it was assumed to be more robust against outliers because and it allowed calculations in reduced time‐segments. Vicon data was processed using Vicon Nexus (Version: 2.10.1. Vicon Motion Systems). In Nexus, the upper limb model was applied for the automatic execution of joint center calculations and to generate kinematic data.^21^ Participant‐specific anthropometric data (e.g., elbow width, shoulder offset) were added to the model in Nexus, whereas the IMU software did not allow model‐individualization. Each joint angle and angular velocity of both systems were derived from XZ′Y″ Euler rotation sequences. In Nexus the XZ′Y″ Euler angle sequences for ROM and PAV/MAV were interpreted as follows: X= “ABD/ADD,” Y= “HFLEX/HEXT”/“ER/IR,” Z= “VFLEX/VEXT.” Since PNF is a multiplanar movement all three components of both systems were used for interpretation (start and end phase for PNF‐X (=ABD/ADD), PNF‐Y (=ER/IR/HFLEX/HEXT), PNF‐Z (=VFLEX/VEXT)). Subsequently, it was exported into Excel‐format followed by the same movement fragmentation and minimum/maximum calculations as previously described. As both methods utilized different recording sampling frequencies (IMU: 147 Hz, Vicon: 500 Hz) outcome files were resampled to a frequency of 200 Hz by reducing Vicon‐ and interpolating IMU data. This step was necessary to match the extracted outcome data sheets from both systems.

### 2.5 Statistical analysis

Descriptive statistical analysis was performed for the anthropometric and kinematic data (mean ± standard deviation [SD]). Criterion validity was assessed by Bland and Altman analysis (bias and limits of agreement (upper LoA: bias +1.96 × SD; lower LoA: bias −1.96 × SD), Intraclass‐correlation coefficient (ICC, using a two‐way mixed model) and root mean square error (RMSE [°] and [°/s]) were calculated for ROM [°], TROM [°], PAV [°/s] as well as MAV [°/s] for all simple and complex shoulder movements between the golden standard (mocap system) and the “off‐the‐shelf sensor‐software solution (IMU) using a statistics software (IBM SPSS statistics; Version: 25.0. IBM Corp). Hereby, the outcomes of the mocap system were used as the reference for the IMU values. Heteroscedasticity of data was assessed by visual inspection of the resulting Bland and Altman plots for all outcome parameters.

## 3 RESULTS

All participants were able to execute the movement tasks. Interpretation of ICC indicators were based on those used in previous investigations: poor (less than 0.5), moderate (between 0.5 and 0.7), good (between 0.7 and 0.9) and excellent (greater than 0.9).^36^ Bland and Altman analysis for the kinematic measures are summarized in Tables 2 and 3 and Supporting Information: Figures 4 and 5 in Supporting Information: File 2.

**Table 2:** Absolute (mean ± SD) ROM and TROM as well as corresponding validity measures.

	Range of motion (ROM)						Total range of motion (TROM)					
Movement	Vicon [°]	IMU [°]	RMSE [°]	ICC	Bias [°]	Upper; lower LoA [°]	Vicon [°]	IMU [°]	RMSE [°]	ICC	Bias [°]	Upper; lower LoA [°]
ABD	160.9 ± 10.9	112.7 ± 12.8	12.2	0.048	48.2	84.5; 11.9	194.0 ± 24.9	155.2 ± 19.9	20.3	0.075	38.7	98.2; ‐20.7
ADD	39 ± 12.9	42.5 ± 12.8	12.8	0.534	‐3.5	24.8; ‐31.8						
HFLEX	114.5 ± 10.5	113.2 ± 15.9	13.0	0.727	1.2	26.0; ‐23.5	145.3 ± 15.1	141.2 ± 21.8	17.6	0.733	4.2	37.9; ‐29.6
HEXT	30.9 ± 10.6	27.9 ± 9.6	9.7	0.296	3.0	28.4; ‐22.4						
VFLEX	157.0 ± 11.3	145.4 ± 15.7	14.0	0.522	11.6	39.2; ‐16.0	195.1 ± 18.9	170.4 ± 21.4	20.3	0.392	24.8	67.9; ‐18.3
VEXT	37.7 ± 10.0	22.4 ± 18.9	17.9	0.369	15.3	49.5; ‐19.0						
ER	65.3 ± 12.8	39.4 ± 10.4	10.7	0.040	25.9	59.3; ‐7.6	122.9 ± 36.9	72.2 ± 15.6	15.9	0.104	50.7	123.5; ‐22.2
IR	65.0 ± 11.5	32.8 ± 12.0	10.4	0.208	32.1	54.1; 10.1						
PNF‐X start	50.1 ± 8.6	42.7 ± 11.9	12.1	0.167	7.4	34.5; ‐19.6	69.3 ± 20.3	60.9 ± 23.8	24.2	0.241	8.5	65.3; ‐48.4
PNF‐X end	19.2 ± 14.8	18.2 ± 14.9	15.0	0.317	1.0	38.2; ‐36.2						
PNF‐Y start	14.4 ± 11.9	12.5 ± 9.9	10.1	0.145	1.8	30.9; ‐27.3	70.2 ± 27.6	53.1 ± 20.5	20.5	0.453	17.1	91.6; ‐57.4
PNF‐Y end	55.8 ± 22.7	40.6 ± 14.5	14.8	0.213	15.2	71.2; ‐40.7						
PNF‐Z start	143.8 ± 31.4	87.9 ± 18.1	15.2	0.259	55.9	106.2; 5.7	171.1 ± 53.9	154.7 ± 36.0	31.1	0.104	50.7	123.5; ‐22.2
PNF‐Z end	27.3 ± 38.7	66.8 ± 25.8	23.3	0.411	‐39.6	28.7; ‐107.9						

abbreviations: range of motion (ROM), total range of motion (TROM), abduction/adduction (ABD/ADD), horizontal flexion/extension (HFLEX/HEXT), vertical flexion/extension (VFLEX/VEXT), external/internal rotation (ER/IR) and a complex movement pattern (PNF X/Y/Z), IMU (inertial measurement unit), root mean square error (RMSE), intraclass‐correlation coefficient (ICC), systematic error (bias) with the limits of agreement (upper LoA: bias + 1.96*SD; lower LoA: bias ‐ 1.96*SD.

**Table 3:** Absolute (mean ± SD) PAV and MAV as well as corresponding validity measures.

	Peak angular velocity (PAV)						Mean angular velocity (MAV)					
Movement	Vicon [°/s]	IMU [°/s]	RMSE [°/s]	ICC	Bias [°/s]	Upper; lower LoA [°/s]	Vicon [°/s]	IMU [°/s]	RMSE [°/s]	ICC	Bias [°/s]	Upper; lower LoA [°/s]
ABD	90.5 ± 22.4	69.0 ± 15.1	15.2	0.202	21.5	69.4; ‐26.4	28.1 ± 9.6	22.7 ± 8.1	7.4	0.562	5.4	23.6; ‐12.8
ADD	91.3 ±18.8	70.0 ± 16.4	14.6	0.447	21.3	56.2; ‐13.6	8.9 ± 3.7	7.3 ± 7.3	7.4	0.235	1.6	16.5; ‐13.3
HFLEX	107.9 ± 25.9	97.6 ± 27.6	18.3	0.865	10.2	46.3; ‐25.9	40.7 ± 24.3	36.1 ± 19.9	15.4	0.786	4.6	41.1; ‐31.9
HEXT	111.2 ± 27.5	89.4 ±24.8	17.6	0.838	21.8	60.2; ‐16.6	21.3 ± 12.3	24.6 ± 10.1	9.9	0.465	‐3.3	22.7; ‐29.3
VFLEX	98.6 ± 44.0	68.8 ± 25.8	21.1	0.571	29.9	98.5; ‐38.8	23.2 ± 12.8	29.7 ± 20.2	20.9	0.056	‐6.4	41.1; ‐53.9
VEXT	93.6 ± 33.2	76.1 ± 27.0	26.7	0.392	17.5	88.9; ‐53.9	8.8 ± 4.9	14.3 ± 7.0	6.1	0.532	‐5.5	6.3; ‐17.3
ER	‐	‐	‐	‐	‐	‐	37.8 ± 13.9	12.5 ± 9.0	8.7	0.187	25.3	51.7; ‐1.0
IR							48.3 ± 13.1	20.5 ± 9.8	9.1	0.198	27.8	52.2; 3.4
PNF‐X start	‐	‐	‐	‐	‐	‐	48.9 ± 27.1	17.3 ± 11.0	10.8	0.214	16.4	106.3; ‐73.6
PNF‐X end							60.5 ± 32.3	52.0 ± 33.8	33.1	0.463	8.4	85.0; ‐68.1
PNF‐Y start	‐	‐	‐	‐	‐	‐	18.0 ± 13.2	29.2 ± 22.0	22.5	0.019	‐11.2	38.8; ‐61.2
PNF‐Y end							19.2 ± 19.5	24.4 ± 17.1	16.2	0.539	‐5.2	35.0; ‐45.4
PNF‐Z start	‐	‐	‐	‐	‐	‐	68.5 ± 37.4	43.7 ± 33.8	34.2	0.235	24.8	115.3; ‐65.7
PNF‐Z end							30.0 ± 24.3	34.9 ± 27.9	26.7	0.538	‐4.9	53.1; ‐62.9

abbreviations: peak angular velocity (PAV), mean angular velocity (MAV), abduction/adduction (ABD/ADD), horizontal flexion/extension (HFLEX/HEXT), vertical flexion/extension (VFLEX/VEXT), external/internal rotation (ER/IR) and a complex movement pattern (PNF X/Y/Z), IMU (inertial measurement unit), root mean square error (RMSE), intraclass‐correlation coefficient (ICC), systematic error (bias) with the limits of agreement (upper LoA: bias + 1.96*SD; lower LoA: bias ‐ 1.96*SD)

### 3.1 ROM

Absolute ROM and TROM as well as ICC, RMSE and bias ± LoA values are stated in Table 2. ICC of ROM analysis ranged between 0.048 and 0.727. Moderate correlations were shown for HFLEX (0.727) and ADD (0.534) as well as VFLEX (0.522), respectively. Overall, TROM assessment resulted in ICC ranges between 0.075 and 0.733, whereas moderate correlations were calculated for HFLEX/HEXT (0.733). RMSE of ROM ranged from 9.7° to 23.3°, the lowest values were calculated for HEXT (9.7°) and for PNF y start (10.1°) movements. Overall RMSE of TROM was reported between 15.9° and 31.1°, whereas the lowest RMSE was demonstrated for ER/IR (15.9°) and HFLEX/HEXT (17.6°). Systematic ROM errors (bias) exhibited ranges between 1.0° and 55.9°, but the lowest values were shown for PNF x start (1.0°) and HFLEX (1.2°), as well as TROM (range: 4.2°–50.7°) HFLEX/HEXT (4.2°) and PNF x start/end (8.5°) between the gold standard and the IMU “off‐the‐shelf” system. Visual analysis of the Bland and Altmann plots revealed homoscedastic distribution of ROM data *(*see supporting Information: File 2 *)*.

### 3.2 Angular velocities

Table 3 summarizes absolute PAV and MAV results as well as ICC, RMSE and bias ± LoA values. Overall ICC PAV analysis ranged between 0.202 and 0.865, whereas good correlations were shown for HFLEX (0.865) and HEXT (0.838). Regarding MAV, ranges between 0.019 and 0.786 were found and good correlations were found for HFLEX (0.786). PAV RMSE resulted in ranges between 14.6°/s and 26.7°/s whereas the lowest RMSE were reported for ADD (14.6°/s) and ABD (15.2°/s). Concerning MAV, ranges between 6.1°/s and 34.2°/s were present and the lowest RMSE were found for VEXT (6.1°/s), as well as ABD (7.4°/s) and ADD (7.4°/s). Systematic errors for PAV exhibited ranges between 10.2°/s and 98.5°/s and were lowest for HFLEX (10.2°/s), as well as MAV ranges of 1.6°/s and 25.3°/s were calculated with lowest values for ADD (1.6°/s) and HFLEX (4.6°/s), respectively. Additionally, for the complex PNF movements, scattered data points in the Bland and Altman plot may be indicating the appearance of heteroscedasticity angular velocity data. During the single‐plane movements a mostly homogeneous variation of data points was evident (see Supporting Information: File 2 *)*.

## 4 DISCUSSION

Portable sensor systems have become a growing field of interest, for professionals performing clinical assessments in multiple rehabilitative settings.^12^, ^29^, ^43^ This study aimed to validate a commercially available “off‐the‐shelf” IMU sensor‐software system for the assessment of shoulder kinematics during single‐ and multiplanar movements. Although IMU systems generally may exhibit sufficient validity for shoulder kinematics, there is considerable variability between studies due to the large amount of customization of software and calibration methods.^44^, ^45^, ^46^ The findings of the present experiment indicate that the investigated “off‐the‐shelf” sensor system achieved the highest but still insufficient accuracy for assessing ROM and angular velocities in the transverse plane, when compared to the gold standard. Nevertheless, more accurate results were expected but not attained for the other single‐plane movements. Contrary to the present investigation, El‐Gohary et al. reported a low RMSE during shoulder ABD and VFLEX (<5.5°).^47^ The authors modified a biomechanical model and the integrated fusion algorithm of the used IMU sensors for the specific task. However, the used kinematic model was applied to a modified inertial tracking algorithm which could reduce magnetic field disturbances and accumulated sensor error drift over time. Comparable values were obtained for shoulder elevation in a recent investigation by Morrow et al. during functional tasks.^35^ The authors fused the IMU sensor data into quaternion rotation matrices before extracting the final Euler angles by using custom software scripts. It could be argued that such cumbersome procedures to assess valid kinematic data may not be accessible for the majority of health professionals. Correspondingly, we aimed to minimize the amount of customization and post‐record processing, deeming this simplification as crucial for the implementation of such mobile systems into clinical practice. We therefore considered our sensor‐software system to be a “black‐box” (=unmodified application) which allows clinical professions to gain real‐time data without further modifications of the software. However, this approach of nonlaboratory data assessment led to major systematic bias for the complex PNF pattern as well as simple shoulder ABD. In this investigation angular velocity outcome parameters were generally more accurate compared to ROM output. Since only gyroscope data was used to extract velocity while ROM was obtained by the internal fusion of all integrated sensors, the general source of error may be identified in the used software rather than the integrated hardware sensors. Within the software it was not reported whether the calibration procedure was successful. Based on that, a thorough investigation whether the IMU sensor axis where in line with the anatomical axis of the shoulder joint was not possible, which has a major impact on the kinematic parameters. Without this and the missing information on the exact sensor placement, the kinematic outcomes may have been influenced by systematic measurement error, which could be supported by Bland‐Altmann analysis. Additionally, the gold standard as well as the IMU software utilized different biomechanical models. The Mocap system is postulated to be more accurate as participant‐specific parameters (e.g., shoulder offset and elbow width) are added to the used model (Vicon upper limb model). Therefore, glenohumeral joint center calculations and the corresponding kinematic data are more precise due to individualization. The IMU software on the other hand utilizes the identical standard parameters for every participant to calculate the rotational displacement between the thorax and the upper arm segment. These basic methodological differences were expected but may not fully explain the differences in accuracy between both systems observed in this investigation. In overhead‐athletes it was shown that side‐to‐side differences of >5° in ER may indicate increased risk of injury.^2^ With the calculated systematic error of 25.9° this clinically important difference is exceeded by far. Therefore, the used commercial “off‐the‐shelf” sensor‐software system may not be mature enough for application in overhead athletes or the general clinical population. Although time‐efficiency was not assessed in this trial, it took a relatively short time (less than 10 min) to prepare each subject, calibrate the IMU system and evaluate all movement conditions. Additionally, the software interface and set‐up procedure were straight forward and easy to use. If the measurement errors were eliminated, the system could allow health professionals to assess multiple dynamic tests without interruptions, whilst gaining and extracting real‐time movement data in the future.

### 4.1 Limitations

A prevalent source of error in body‐worn sensor systems lies in soft tissue artifacts, especially in the frontal and sagittal planes.^48^ Errors due to muscle tension, sensor tilt and rotation were noticeable in this investigation, which may have led to biased data peaks or the inability to recognize motion. This problem may be solved by attaching the sensors on bony landmarks at the elbow or by using skin‐tight circular straps like those used in a similar investigation.^36^ However, the exact sensor positioning was not provided accurately enough in the software. In addition, magnetic disturbances within the measurement volume may have limited the precision of the used IMU sensor‐software system. Although calibration procedures included the magnetometers, sudden changes in the laboratory environment may not be omitted. This may also explain the relatively large amount of corrupted motion outputs in several trials. Following the calibration procedures, it was not possible to track whether the alignment of the sensors axis and the anatomical axis was successful, which potentially biased all outcomes. In this regard, repeated measurements for reliability assessment may have delivered viable information on time‐ and measurement dependent sources of error. Overall, the greatest limitation of commercially available systems is that the integrated sensor fusion as well as output software cannot be edited by the investigator. This makes it generally easier to handle for clinicians but prohibits further interventions concerning individualization or task specifications.

## 5 CONCLUSIONS

Overall, limited agreement was evident between the portable “off‐the‐shelf” sensor‐software system and the gold standard (Mocap). Although the overall criterion validity may not be sufficient yet, it is important to understand that commercially available and applicable automatic processing software might be particularly important for the professionalization of therapy and training practices. Further research is necessary to investigate whether modified “off‐the‐shelf” mobile sensor‐software systems are accurate enough to assess clinically important adaptations in shoulder kinematics for athletic and clinical populations.

## AUTHOR CONTRIBUTIONS

**Jakob Henschke**: Conceptualization; data curation; formal analysis; investigation; methodology; project administration; validation; writing—original draft; writing—review and editing. **Hannes Kaplick**: Conceptualization; data curation; formal analysis; investigation; methodology; supervision; validation; writing—review and editing. **Monique Wochatz**: Conceptualization; investigation; supervision; writing—original draft; writing—review and editing. **Tilman Engel**: Conceptualization; data curation; formal analysis; investigation; methodology; project administration; supervision; validation; writing—original draft; writing—review and editing.

## CONFLICT OF INTEREST

The authors declare no conflict interest.

## TRANSPARENCY STATEMENT

The lead author affirms that this manuscript is an honest, accurate, and transparent account of the study being reported; that no important aspects of the study have been omitted; and that any discrepancies from the study as planned (and, if relevant, registered) have been explained.

## Supporting information

Supplementary information.

Supplementary information.

## Data Availability

The data that support the findings of this study are available from the corresponding author upon reasonable request.
