# Erratum

**DOI:** 10.1002/hsr2.992

**Published:** 2022-12-12

In the article by Henschke et al.,^1^ the funding information needs to be appended in the acknowledgment section:

Funded by the Deutsche Forschungsgemeinschaft (DFG, German Research Foundation)—Projektnummer 491466077.

We apologize for the error.
