# Supplementary material for: Assessing the validity of inertial measurement units for shoulder kinematics using a commercial sensor‐software system: A validation study
Source: Health Sci Rep. 2022 Aug 10;5(5):e772. doi: 10.1002/hsr2.772 (PMC9364332; doi:10.1002/hsr2.772)
Supplement: Supplementary file 1 — Supplementary information. [file HSR2-5-e772-s001.docx]

Figure. 3. Measuring positions and movement phases.

| **Start** | **Abduction** | **Adduction** |
| --- | --- | --- |
| 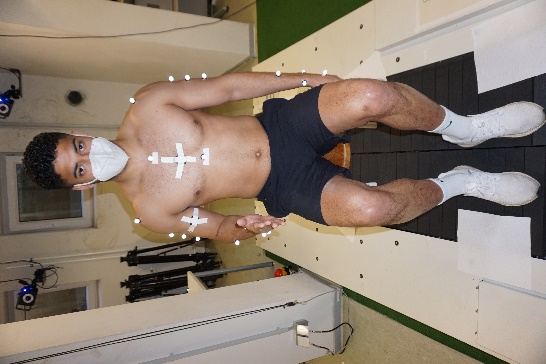 | 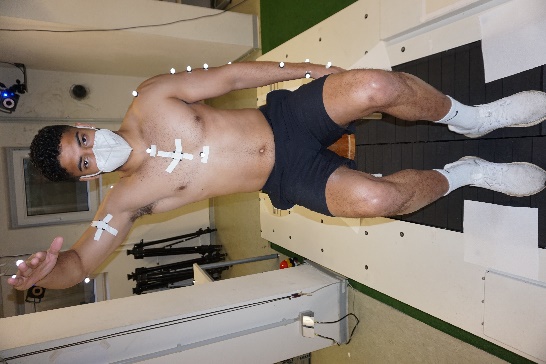 | 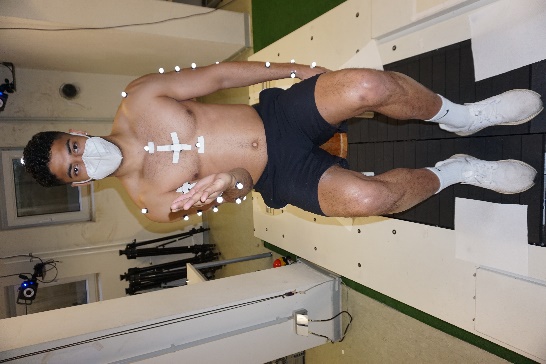 |
| **Start** | **Horizontal flexion** | **Horizontal extension** |
| 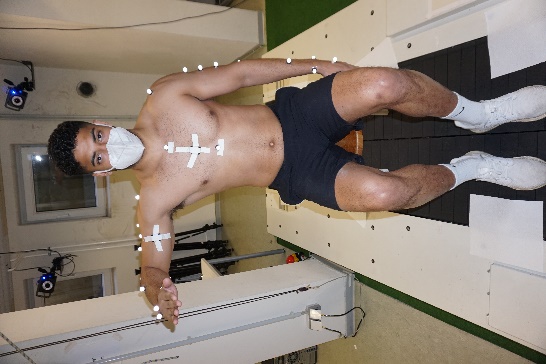 | 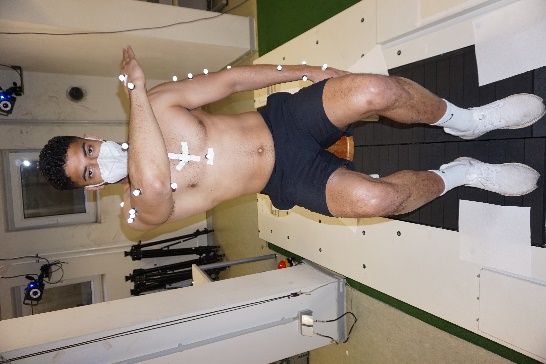 | 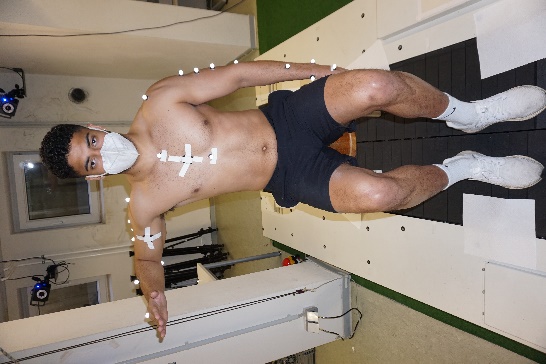 |
| **Start** | **Vertical flexion** | **Vertical extension** |
| 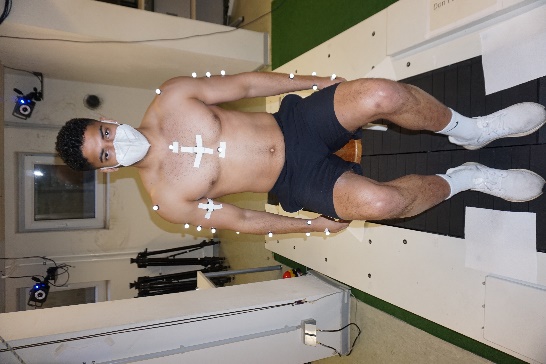 | 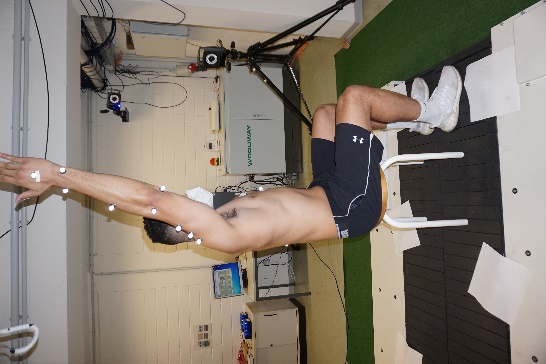 | 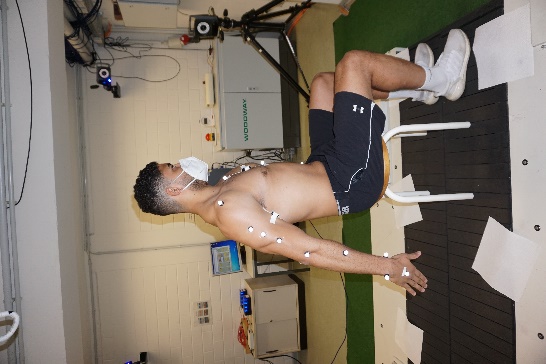 |

| **Start** | **External rotation** | **Internal rotation** |
| --- | --- | --- |
| 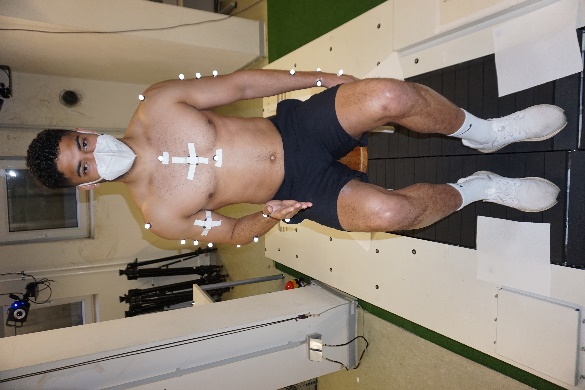 | 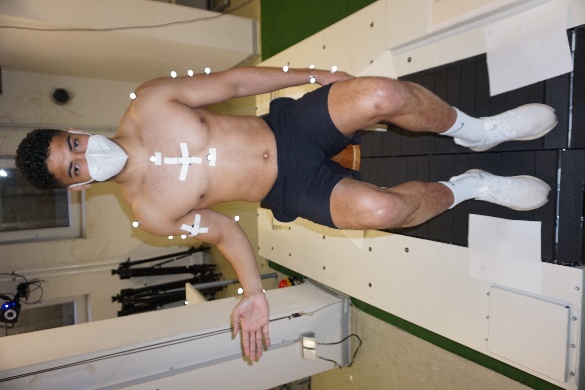 | 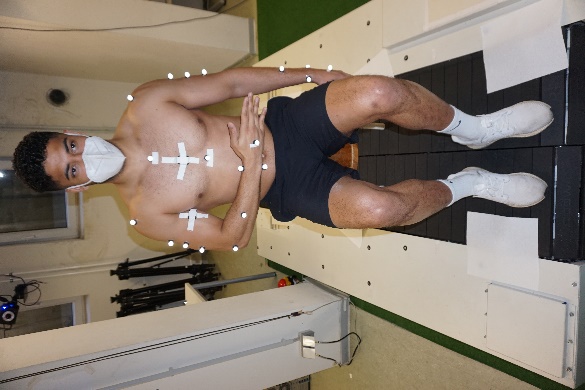 |
| **Start** | **PNF-start** | **PNF-end** |
| 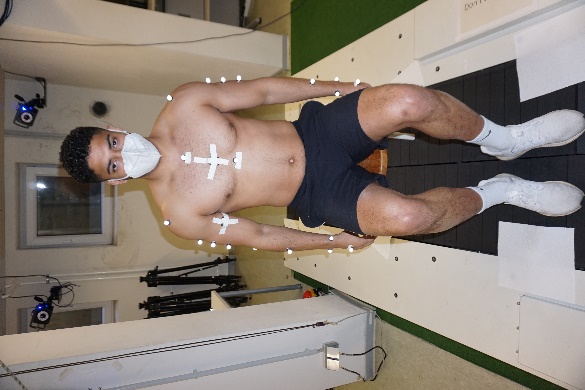 | 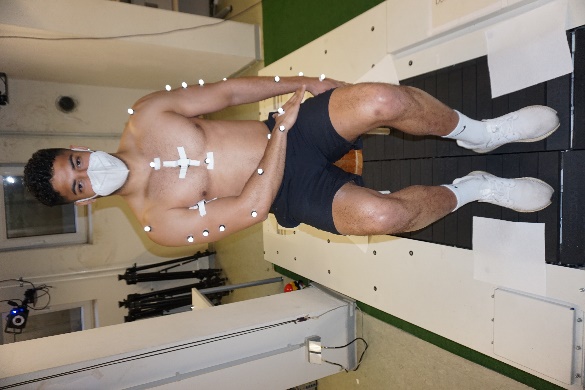 | 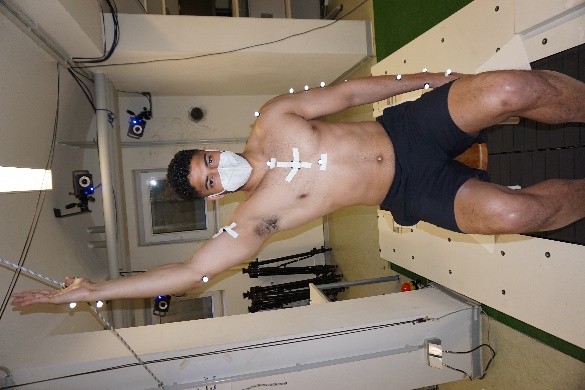 |

Abbreviations: PNF= complex movement pattern (divided in start/end and X/Y/Z Euler angle components). Movements were executed in the following order: starting position (first column from left), to first terminal position (middle column) to second terminal position (right column) and returning to starting position (first column from left) for all simple and complex shoulder movements.
