# Supplementary material for: Assessing the validity of inertial measurement units for shoulder kinematics using a commercial sensor‐software system: A validation study
Source: Health Sci Rep. 2022 Aug 10;5(5):e772. doi: 10.1002/hsr2.772 (PMC9364332; doi:10.1002/hsr2.772)
Supplement: Supplementary file 2 — Supplementary information. [file HSR2-5-e772-s002.docx]

Figure 4. Bland-Altmann plots for ROM [°] and TROM [°] during single- and multiplanar movements.

| **Abduction/adduction** | **Horizontal flexion/extension** | **vertical flexion/extension** |
| --- | --- | --- |
| **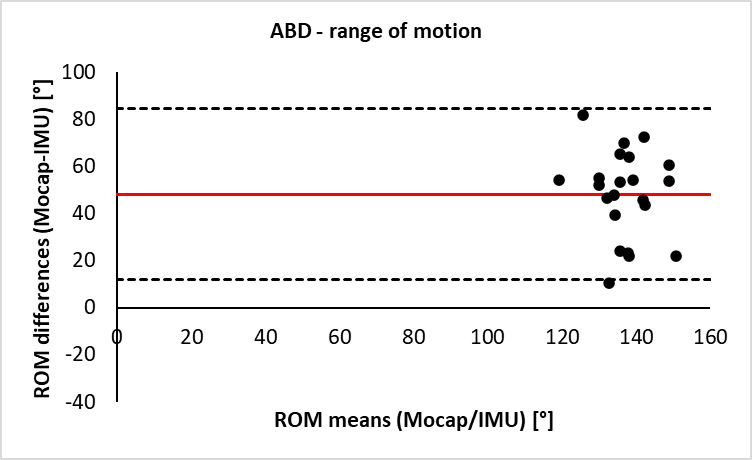** | **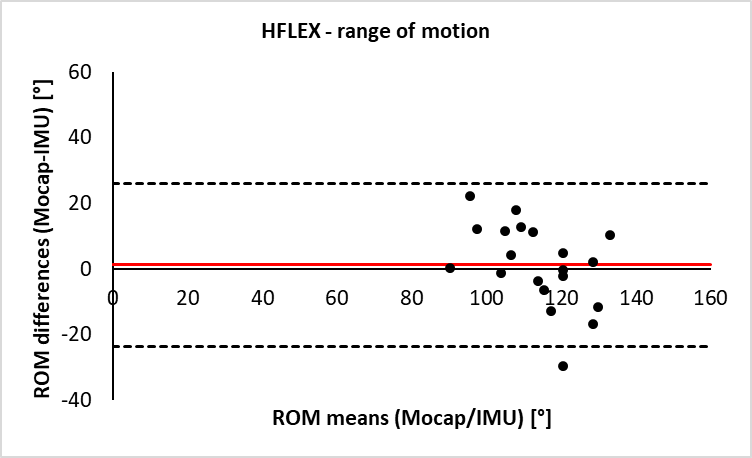** | **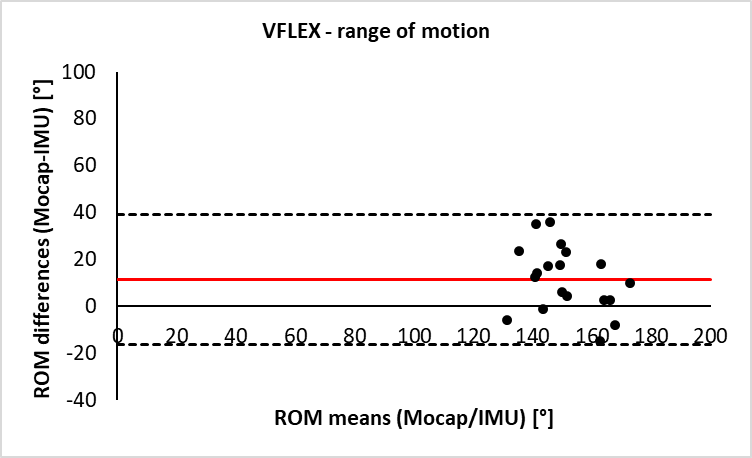** |
| **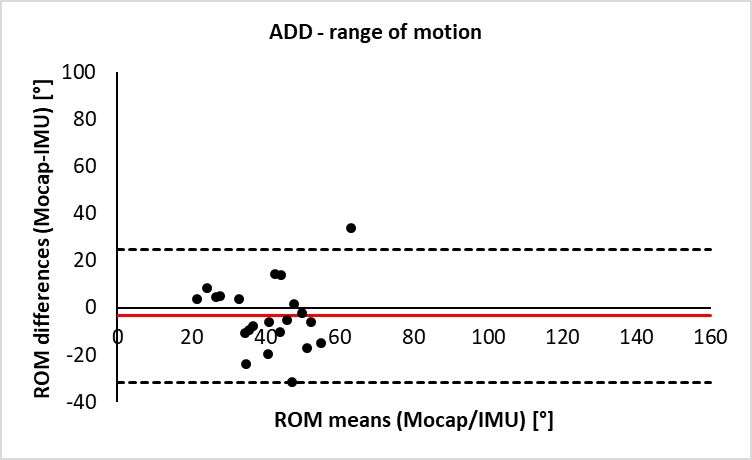** | **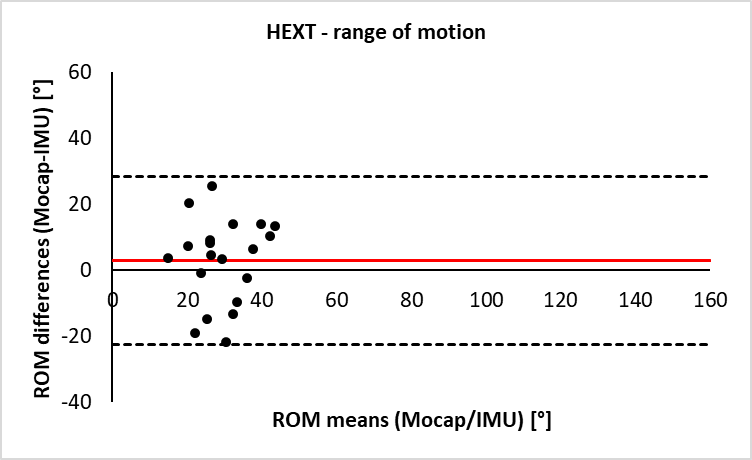** | **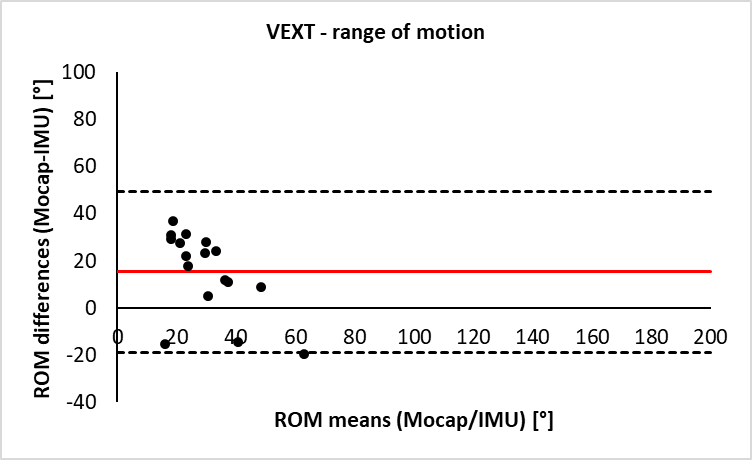** |
| **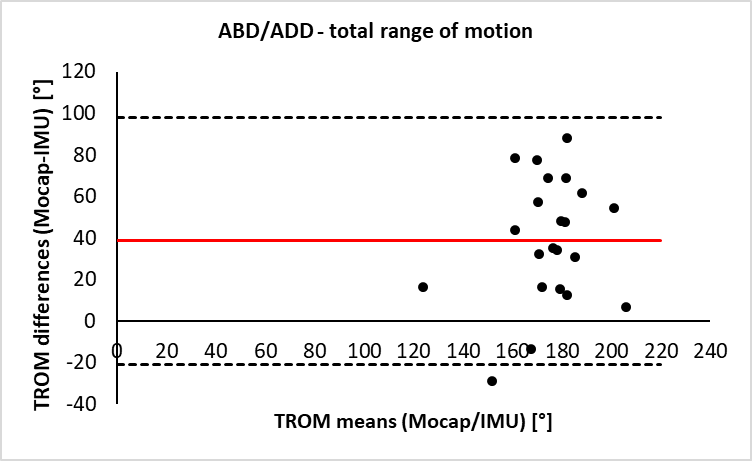** | **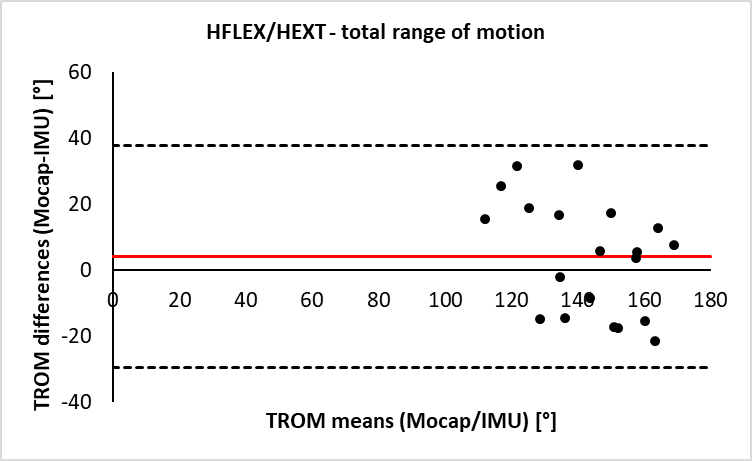** | **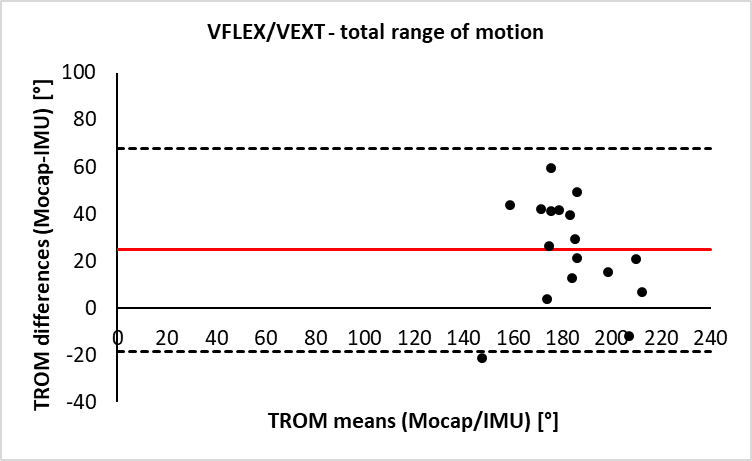** |

Figure 4. Bland-Altmann plots for ROM [°] and TROM [°] during single- and multiplanar movements (continued).

| **External/internal rotation** | **PNF-X start/end** | **PNF-Y start/end** |
| --- | --- | --- |
| **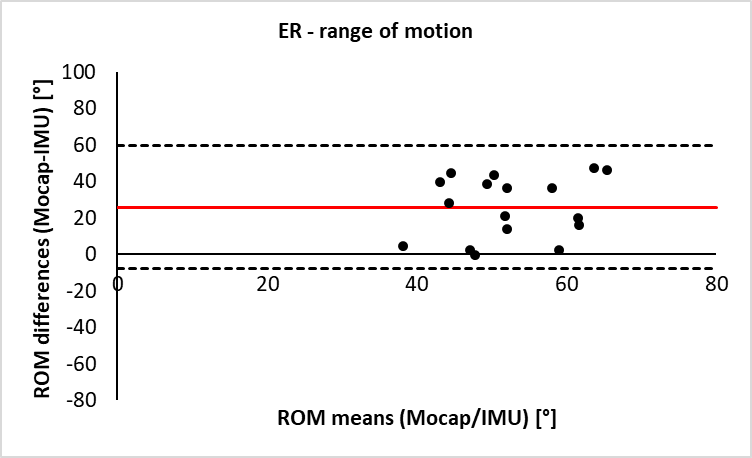** | **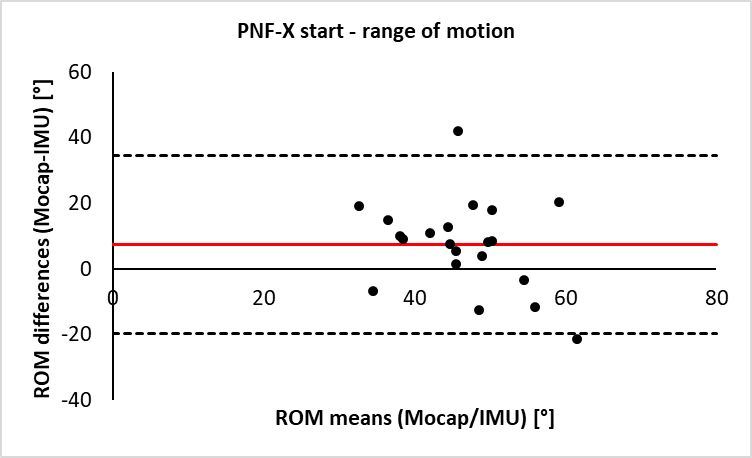** | **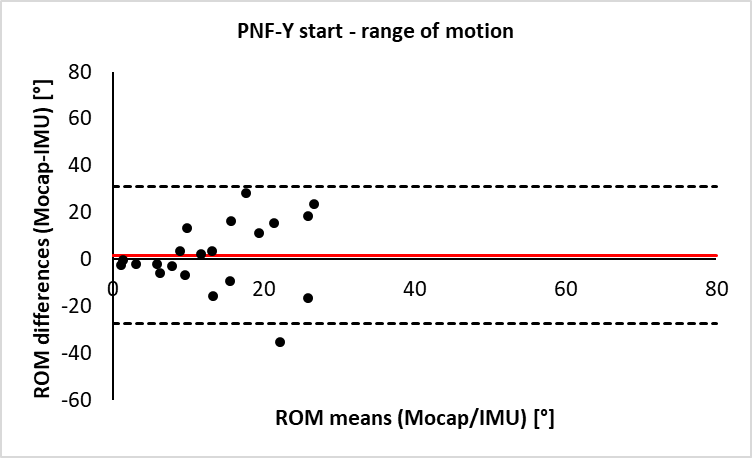** |
| **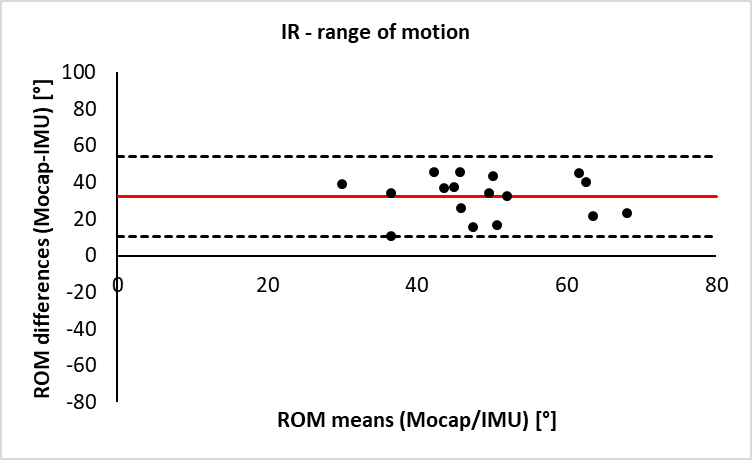** | **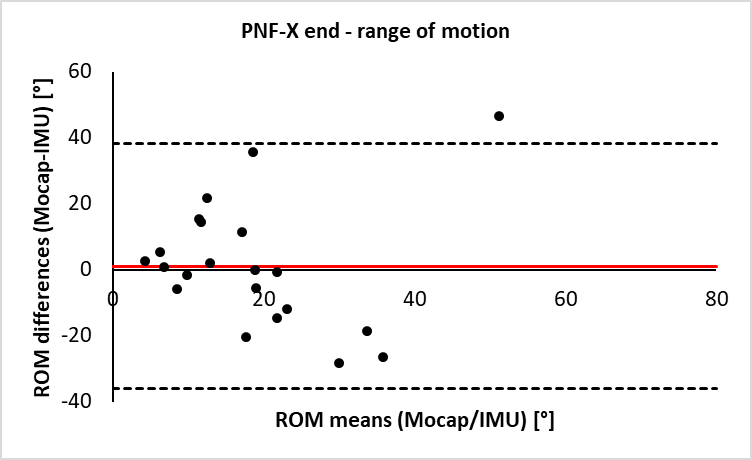** | **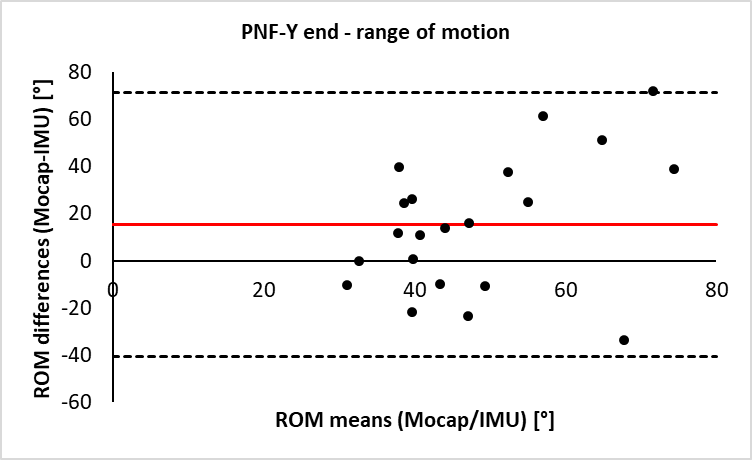** |
| **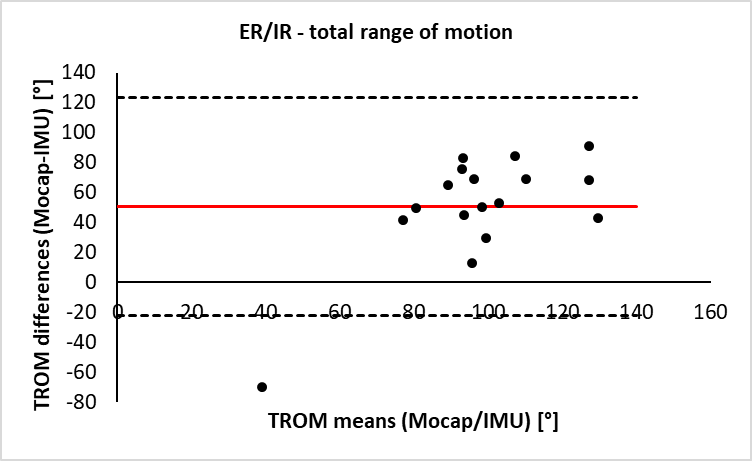** | **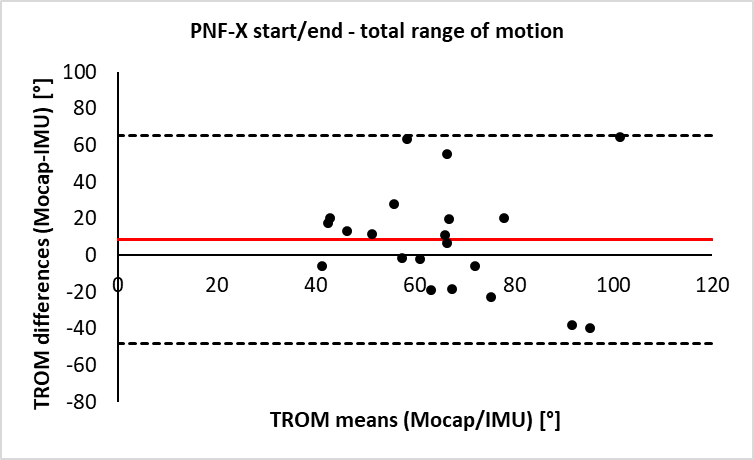** | **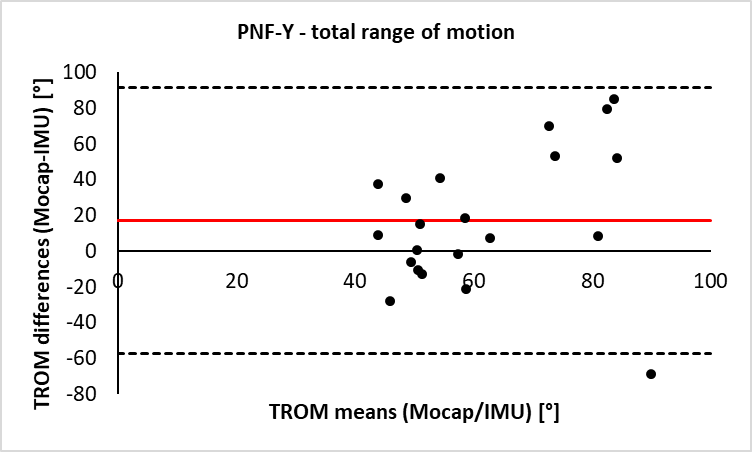** |

Figure 4. Bland-Altmann plots for ROM [°] and TROM [°] during single- and multiplanar movements (continued).

Abbreviations: ROM= range of motion, TROM= total range of motion, Mocap= motion capture system, IMU= inertial measurement unit, ABD=abduction, ADD= adduction, VFLEX= vertical flexion, VEXT= vertical extension, HFLEX= horizontal flexion, HEXT= horizontal extension, ER= external rotation, IR= internal rotation, PNF= complex movement pattern (divided in start/end and X/Y/Z Euler angle components). The red lines show the systematic error (bias), while the black dotted lines indicate the upper (bias + 1.96*SD) and lower limits of agreement (lower LoA: bias - 1.96*SD).

| **PNF-Z start/end** | **PNF-Z start/end** | **PNF-Z start/end** |
| --- | --- | --- |
| **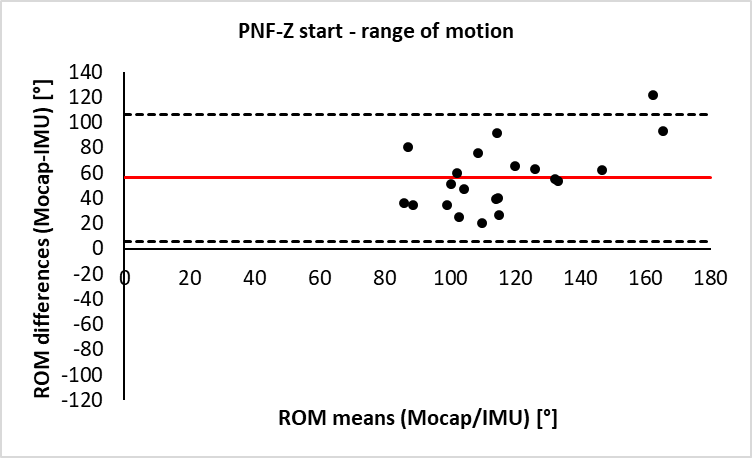** | **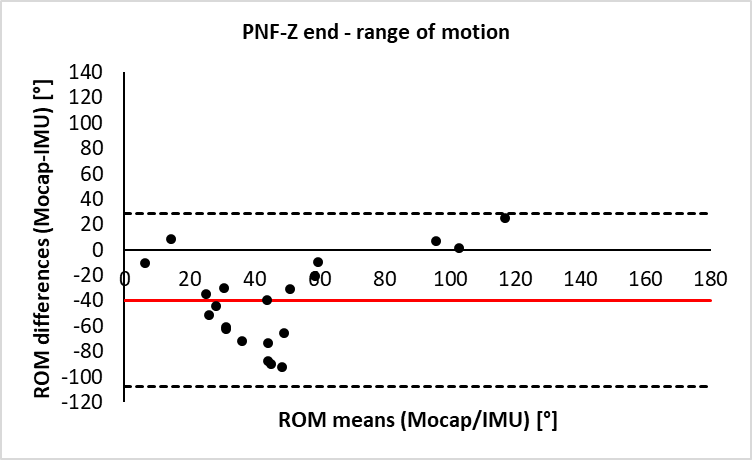** | **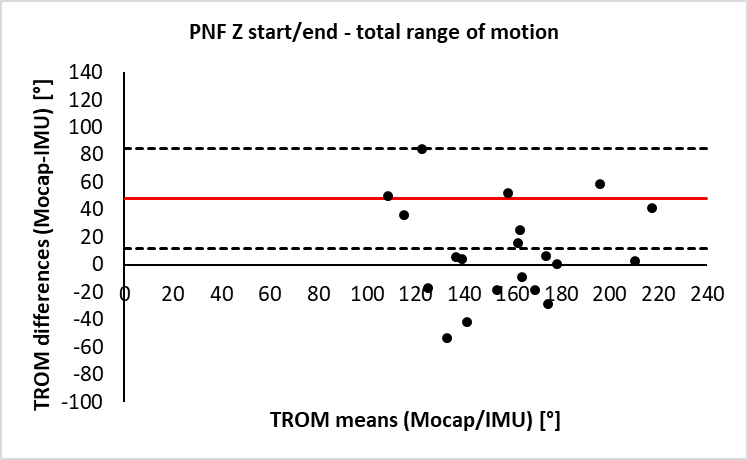** |

Figure 5. Bland-Altmann plots for PAV [°/s] and MAV [°/s] during single- and multiplanar movements.

| **Abduction/adduction** | **Horizontal flexion/extension** | **Vertical flexion/extension** |
| --- | --- | --- |
| **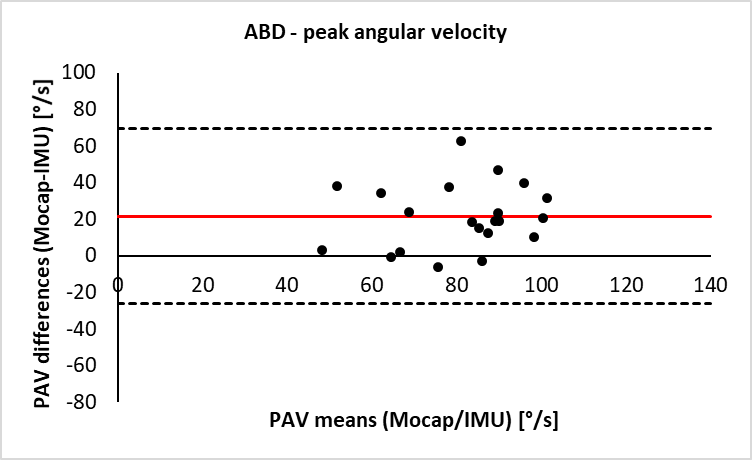** | **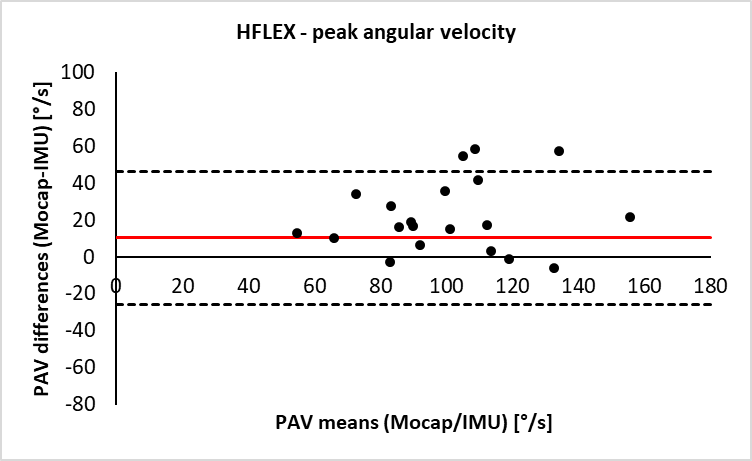** | **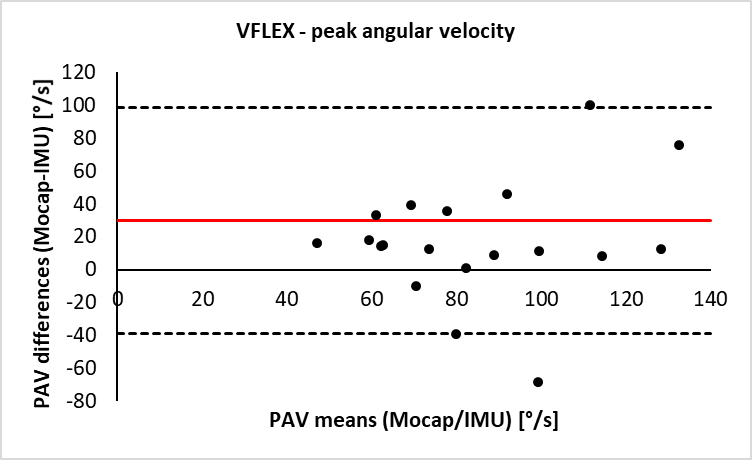** |
| **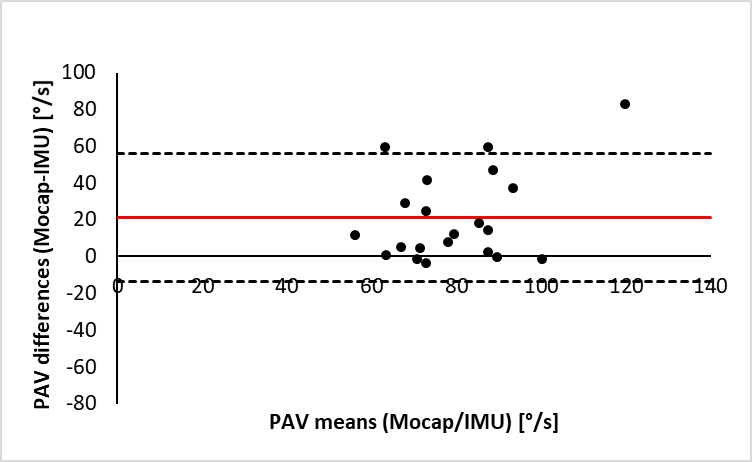** | **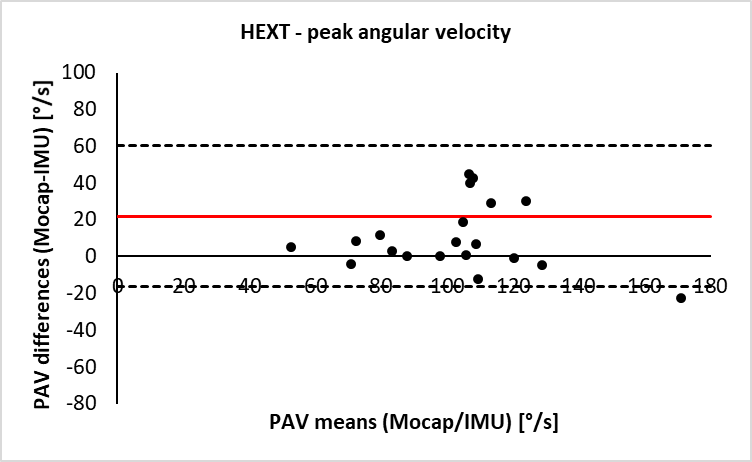** | **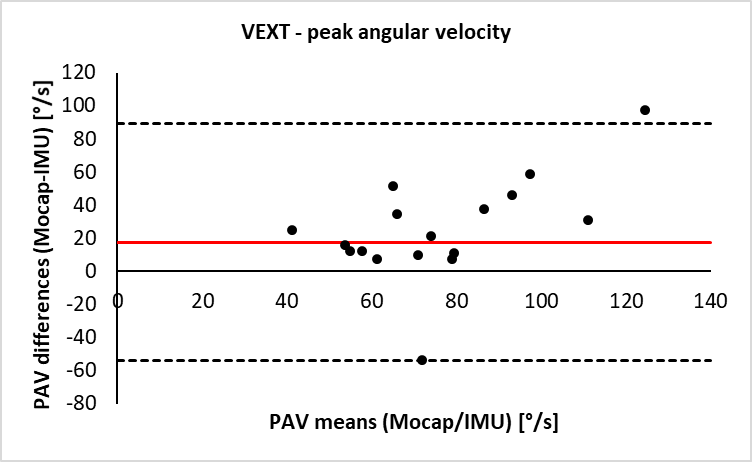** |
| **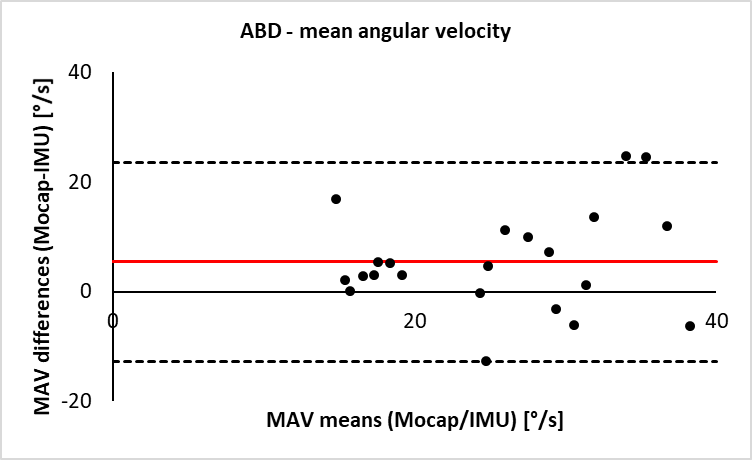** | **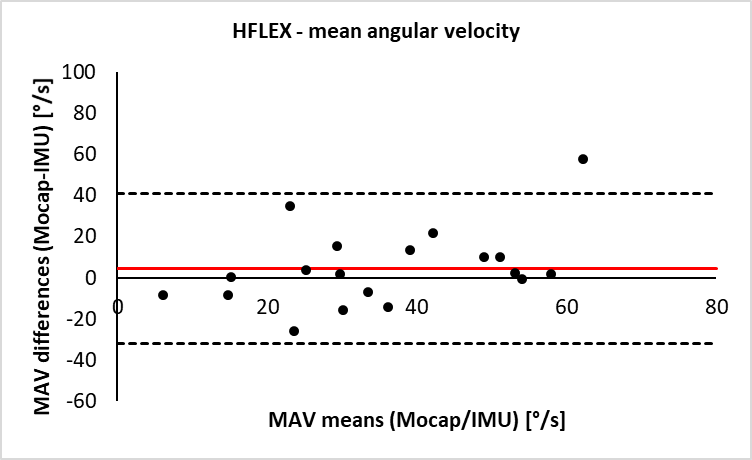** | **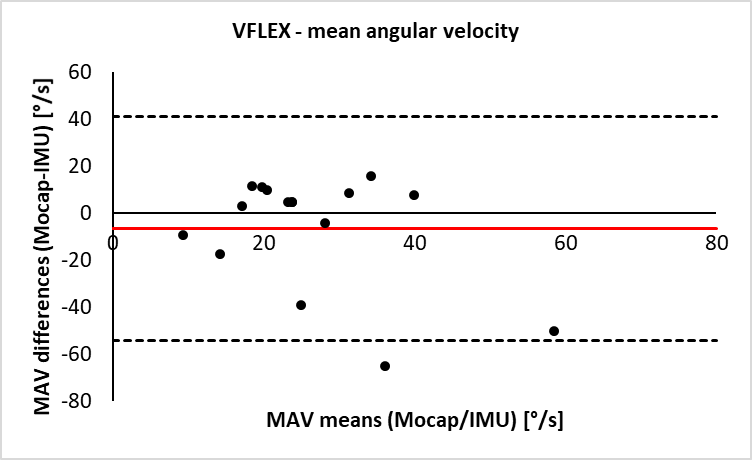** |

Figure 5. Bland-Altmann plots for PAV [°/s] and MAV [°/s] during single- and multiplanar movements (continued).

| **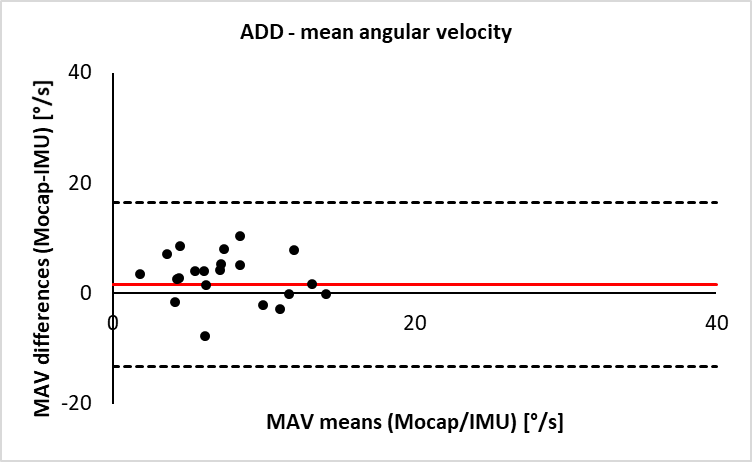** | **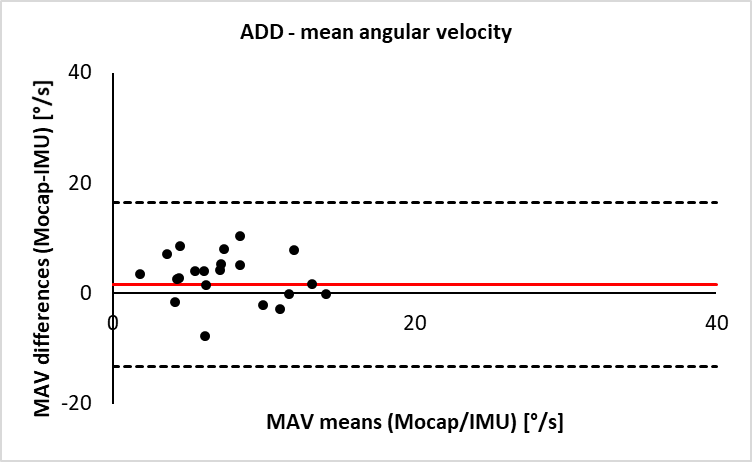** | **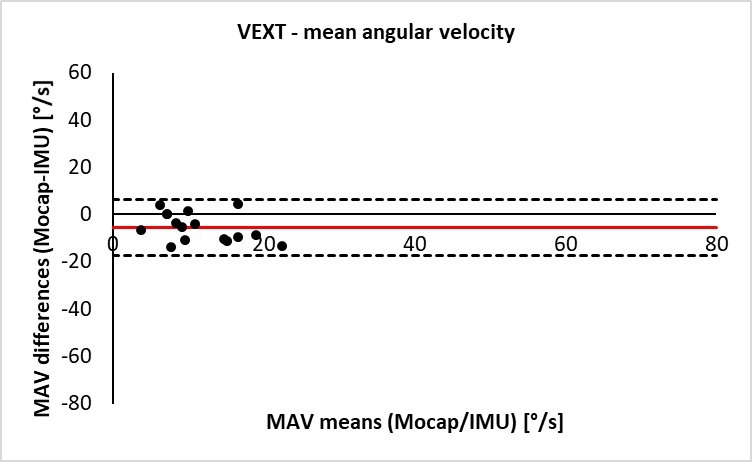** |
| --- | --- | --- |
| **External/internal rotation** | **PNF-X start/end** | **PNF-Y start/end** |
| **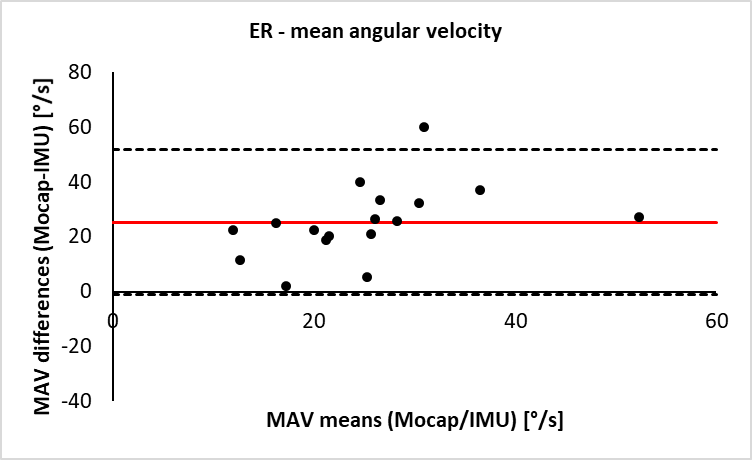** | **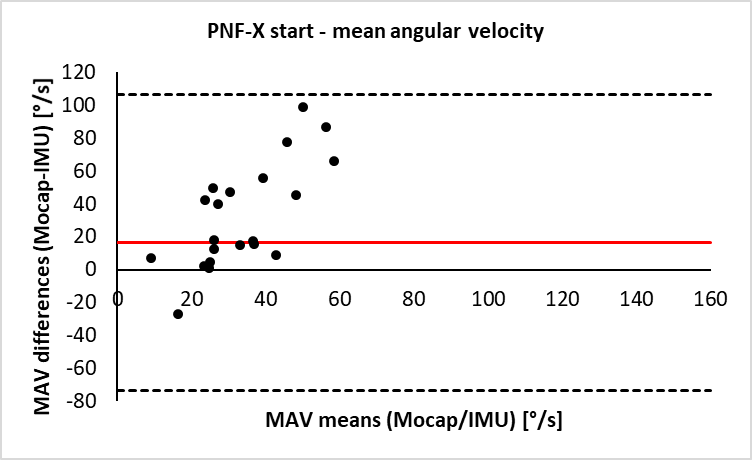** | **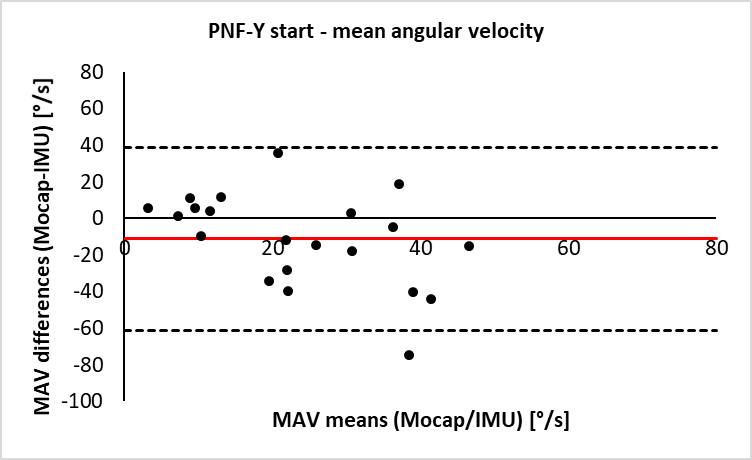** |
| **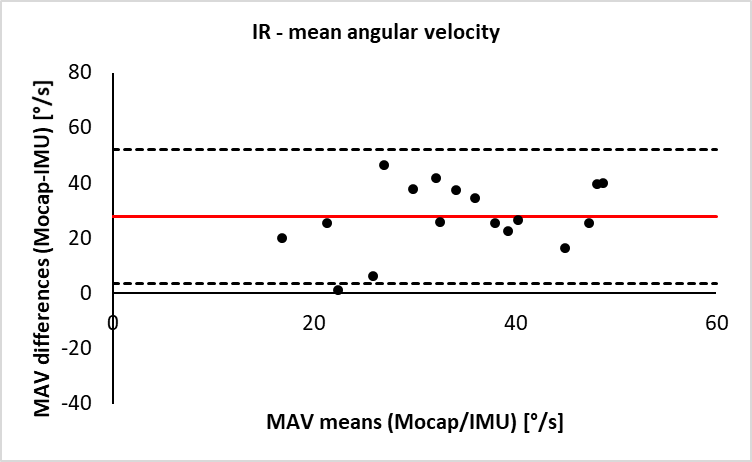** | **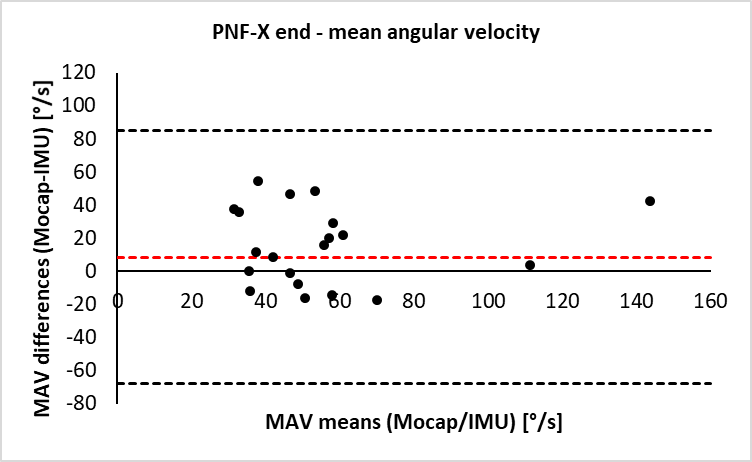** | **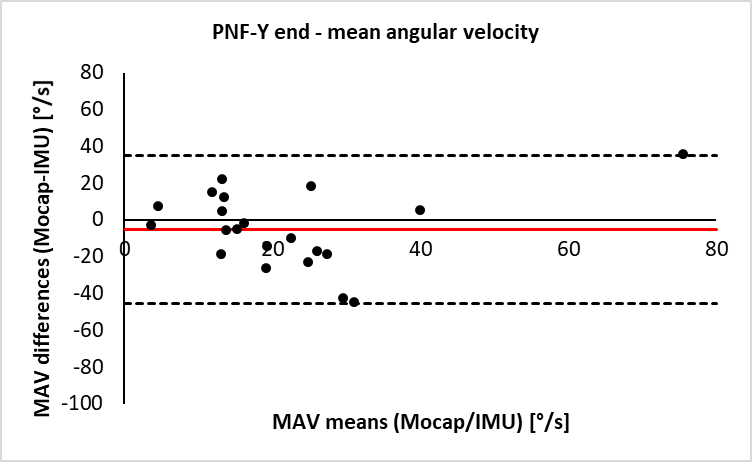** |

Figure 5. Bland-Altmann plots for PAV [°/s] and MAV [°/s] during single- and multiplanar movements (continued).

| **PNF-Z start** | **PNF-Z end** |  |
| --- | --- | --- |
| **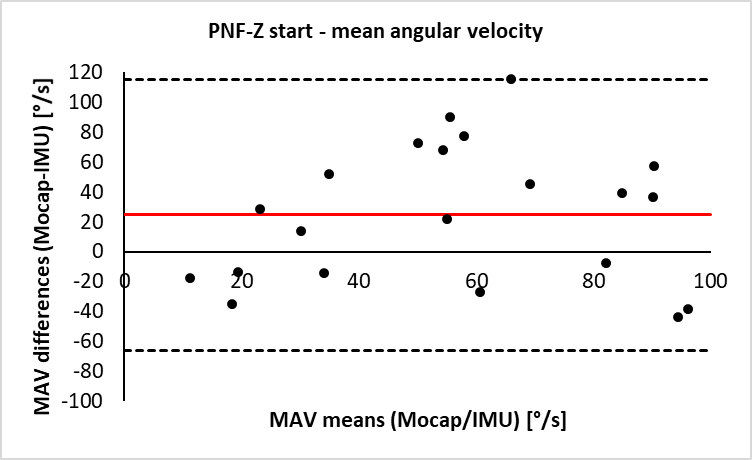** | **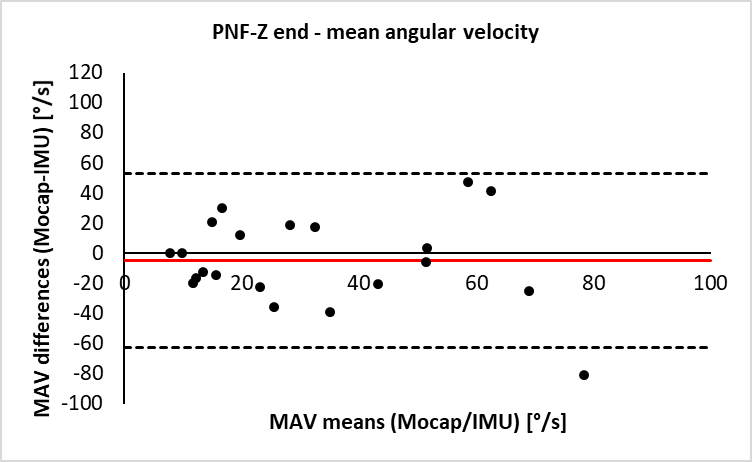** |  |

Abbreviations: PAV= peak angular velocity, MAV= mean angular velocity, Mocap= motion capture system, IMU= inertial measurement unit, ABD=abduction, ADD= adduction, VFLEX= vertical flexion, VEXT= vertical extension, HFLEX= horizontal flexion, HEXT= horizontal extension, ER= external rotation, IR= internal rotation, PNF= complex movement pattern (divided in start/end and X/Y/Z Euler angle components). The red lines show the systematic error (bias), while the black dotted lines indicate the upper (bias + 1.96*SD) and lower limits of agreement (lower LoA: bias - 1.96*SD).
